# The Hospital. Nursing Section

**Published:** 1905-06-03

**Authors:** 


### The Hospital.

### Nursing Section.

Contributions for this Section of "The Hospital" should be addressed to the Editor, "The Hospital" Nursing Section, 28 & 29 Southampton Street, Strand, London, W.C.

No. 975.—Vol. XXXVIII. SATURDAY, JUNE 3, 1905.

### Notes on News from the Nursing World.

### THE NEW MILITARY HOSPITAL.

Although the matron of the new Military Hospital at Millbank has been appointed some little time, and the building itself has the appearance of being finished, with convalescent soldiers lying out on the balconies, it will be some months before the nursing arrangements are anything like complete. It is true that the matron is in residence, and that a certain number of nurses and orderlies are in attendance on the patients already in the hospital, but until the formal inauguration, which it is hoped may be honoured by the presence of Royalty, no visits of inspection will be permitted. There is a very natural desire that when the hospital, which will be one of the most important in the kingdom, owing to the Tropical School of Medicine attached to it, is opened, everything shall be in the best possible order, and this, of course, is not practicable until the workmen are out of the way.

### ADMIRAL ROJDESTVENSKY AND THE SISTER.

A story is told of Admiral Rojdestvensky, whose terrible defeat is the talk of the world this week, which shows how literally he insisted on obedience to his orders. While the Russian fleet was in the waters of Madagascar instructions were issued by the Admiral that after sundown no officer or other person should be on any vessel but his own. One evening a sister of mercy from the *Orel* stayed a considerable time longer, and when she at length wished to retire a steam launch was lowered, and three officers accompanied her back to the hospital ship. The Admiral, hearing of this, at once signalled that the launch was to come alongside the flagship, when he saw the three officers and the sister of mercy. He then gave orders that the nurse was to be escorted to her ship, she, of course, escaping punishment, except her own self reproaches, but the three officers were the next day sent back to Russia in disgrace.

### SECOND THOUGHTS OF THE DUCHESS.

It was announced last week that a committee had been formed in connection with the Lay Association for Promoting the State Registration of Nurses. This is the movement which originated at a drawing-room meeting under the auspices of the Society for the State Registration of Trained Nurses, whose annual gathering on Friday seems to have been devoid of interest. On Monday the Duchess of Montrose, whose name was given as a member of the committee, intimated that since reading the evidence given before the Select Committee of the House of Commons she had decided to withdraw her name. She added: "I feel strongly that different grades of nurses are necessary to meet the requirements of different forms of illness, and that State registration would act detrimentally on those nurses who are not fully trained but are yet doing useful work. As an instance of the lengths to which some of those in favour of State registration are prepared to go, I need only quote the preposterous proposal made by Sir Victor Horsley in his evidence before the Select Committee, namely, 'that he would make it a penal offence for any woman to nurse for gain unless she had received three years' training in a hospital.'" We rejoice that the Duchess of Montrose has had the courage to withdraw so promptly from a position which she found untenable.

### THE SECTARIAN SPIRIT IN DISTRICT NURSING.

The first annual meeting of an organisation called the Chester Free Church Council Nursing Society has just been held. It appears to have had its origin in the fact that one of the rules of the District Nursing Association in the city is that members of the nursing staff must be members of the Church of England. We regret alike the existence of such a rule and the formation of a second society, whose sectarian character is frankly signified by its title. Nursing the sick is not a matter on which the issues of Church or Dissent ought to be raised, and we are sorry that in Chester it should be considered necessary to run two organisations avowedly working for the same excellent ends. This, indeed, is the view which the Sheriff, who presided at the meeting, expressed, and he had the good sense to propose that steps should be taken to join the two forces on a basis that would be satisfactory to both. We hope that the Chester people will have the wisdom to relieve the city from the stigma which must inevitably attach to it so long as its citizens cannot find a common platform on a question which, in nearly every other part of the kingdom, is treated in an entirely non-sectarian spirit.

### CORONATION FUND FOR IRISH NURSES.

The first annual meeting of King Edward the Seventh's Coronation National Fund for Nurses in Ireland was held on Wednesday last week. The report showed that 171 nurses and probationers have joined, and that the Fund has now a capital of £4,475, producing 2½ per cent. Sir Francis Cruise and Sir Frederick Falkiner congratulated the meeting on this result of the movement. The former described it as an insurance and benefit society, while the latter expressed the hope that it would be so extended as to enable the managers to adopt an annuity society. At present the prospects do not seem very exhilarating. It is true that a grant of £10 was made to each of two nurses last year, but an income of £103 is a very small nucleus for an organisation which aspires to be a great benevolent association.

### NURSING AND GARDEN PARTIES.

Lady Dillwyn Llewelyn has set an excellent example early in the summer. In her capacity of president of the penny collecting scheme which was lately organised in order to bring in badly-needed funds for the benefit of the Swansea Nursing Association, she invited to her lovely gardens at Penllergare the 50 secretaries of the branches, and 30 collectors who have undertaken to promote the movement. The whole of the large party drove in brakes from Swansea, and spent the afternoon in the grounds, with the result that they started on their homeward journey encouraged, by friendly social intercourse, to persevere in their efforts. Such gatherings might be held during the next few months with great advantage. They are the cause of pleasure to individuals, and they materially tend to strengthen the *esprit de corps* which does so much to keep a nursing association flourishing.

### THE DIFFICULTY AT PLYMOUTH.

After all, there is to be no Local Government Board inquiry into the friction at Plymouth Workhouse Infirmary. It will be remembered that the Guardians called on the Superintendent Nurse to resign but formulated no specific charges against her. Miss Holliday declined to accede to their request, and replied that she had been commended for the manner in which she has discharged her duties during the past four years, and was able to point out that the Guardians had increased her salary. The Guardians then appealed to the Local Government Board for an inquiry and set out a statement of their case. Thereupon the Superintendent Nurse submitted facts to prove that the friction could not be wholly attributed to her, and the Local Government Board have at length intimated to the Guardians that no prima facie case was made out against the Superintendent Nurse which would justify the holding of an inquiry. This decision does not seem to have been wholly unexpected, and it led to the suggestion by one of the Guardians: "Let us bury the hatchet now, and forget all about the business." Since the last meeting, Mr. Preston Thomas, one of the Local Government Board inspectors, has met the Hospital Council, and, we understand, indicated the weakness of the Guardians' case. He also promised to have an informal chat with the Superintendent Nurse on the difficulties, and we hope that the result will be the cessation of all friction. Miss Holliday, having carried the day, can afford to give as well as take, and the Guardians, as sensible men, will doubtless recognise the wisdom of working in harmony with her.

### THE CUSTOM OF PAYMENT.

At Wakefield County-court last week a question of much interest to nurses engaged in private work was raised. A trained nurse sued a tradesman in the city for the sum of £3 4s., which she stated was the balance due to her for holding herself a month in readiness to attend upon the defendant's wife. The defence was that there was no liability until the nurse actually entered upon her duties. The plaintiff, however, called witnesses to prove that it is customary to pay nurses from the time they have been engaged to keep themselves in readiness, and the case was decided in her favour. It stands to reason that a nurse, like any other person, should be paid from the moment her services are retained. Otherwise, she would naturally decline to engage herself until she was actually wanted.

### BIRMINGHAM INFIRMARY UNDERSTAFFED.

The Birmingham Guardians have under consideration a proposal to extend the nurses' accommodation at the infirmary. At their last meeting it was stated that the infirmary has been understaffed for some time, and that, while additional nurses are badly required, there is no room for more. The infirmary committee desire to add 28 bedrooms at a cost of £3,500. This moderate outlay was opposed on the ground of short notice rather than on principle, and the matter has therefore been referred back for further information. We cannot believe, however, that a body of such enterprise as the Birmingham Guardians will decline to adopt a course which is so clearly requisite in the interests of this admirable training school. That Birmingham Infirmary should pass the unenviable reputation of lagging behind the times is almost inconceivable.

### PROPOSED NEW DEPARTURE AT NORWICH.

The feature of the annual meeting of the Norwich District Nurses' Association, which was held last month at the Deanery, was the speech of the Mayor, who urged the desirability of establishing a provident branch in connection with the home belonging to the association. Assuming that respectable working people do not like to depend upon charity, he said that he saw no reason why the provision of skilled nursing should not be organised on the same lines as friendly societies, and he suggested a subscription of 1s. a year, with a charge of a penny per visit from the nurse to any member of the family of a subscriber. He calculated that the subscriptions of 2,000 members would suffice to pay the salary of a nurse in a large centre, and while admitting the difficulty which might be experienced in obtaining such a number, he insisted that perseverance would be rewarded by success. No decision was arrived at in the matter, but subsequently, after the report had been adopted, the Mayor promised to advocate co-operation between friendly societies and the association. It will be interesting to see the outcome of his efforts.

### DEATH OF LADY BLOOMFIELD.

We regret to record the death of Lady Bloomfield, the founder of the Trained Nurses' Annuity Fund, which occurred on Sunday last, May 21st, after a long illness. The honorary secretary of the Fund, Dr. A. Ogier Ward, writing to us on the subject, says:—"It would not be fitting that she, who first framed a means of providing for nurses after they ceased to be able to provide for themselves, should pass away without some mention in the columns of the chief paper devoted to nurses' interests. Nurses ought to have it brought home to them what a friend they have lost. It is impossible now to say exactly how many have profited by her thought, for the society's early records were lost years ago, but within the last 10 years 20 or more destitute and brokendown nurses have benefited, and 13 are now regularly receiving annuities which they owe to Lady Bloomfield's work. Most grateful and touching letters have come to hand within the last week from nurses who owe protection from the workhouse to these annuities."

### HOSPITAL NURSES FOR ASYLUM WORK.

Two appointments which we announce to-day are significant. The new head nurse and the night superintendent at Roxburgh District Asylum are both hospital-trained nurses. The former, Miss McCloskey, was trained at Sheffield Royal Infirmary, has done private nursing for two years, had three and a half years' experience in a Glasgow surgical home, been sister at her training school, and sister and night superintendent at the General Infirmary, Macclesfield. The latter, Miss Keaghey, was trained at Meath Hospital, Dublin, and has been Queen's nurse for a year and a half, as well as charge nurse for four years and a half at Craiglockhart Mental Hospital.

### UNIVERSITY COLLEGE HOSPITAL LADIES' ASSOCIATION.

The annual general meeting of University College Hospital Ladies' Association took place on Thursday last week, the Duchess of Devonshire in the chair. The hon. secretary, Mrs. M. Bradford, read the annual report, and Mrs. Godlee, the hon. treasurer, the balance sheet. The adoption of the report was moved by Lady Russell Reynolds, and seconded by Viscountess Hampden. It transpired that Mrs. Bradford had provided in one month 61 night-dresses, a most welcome store; for, as the matron explained, fewer garments for women are received than for men, and at present there is great need of the former, especially for female patients going to convalescent homes. Mrs. J. E. Blomfield moved and Mrs. Henry Lucas seconded a resolution altering one of the rules. Votes of thanks to the Hospital Committee for lending the ward for the meeting, and to the Duchess of Devonshire for presiding, terminated the proceedings.

### NURSES AND MISSIONARY WORK.

The third annual meeting of the Nurses' Missionary Union was held at University Hall, Gordon Square, on Thursday last week, from three to nine. Strong emphasis was laid by the speakers on the great opportunities of Christian service which the nursing profession affords, especially in the foreign mission field. Dr. Lankester stated that at the present moment the Church Missionary Society alone has 10 vacant posts waiting for trained nurses. In the report of the Union a list is given of all nurses now working abroad under all the British Missionary Societies, and the total number is about 140. The committee for 1905-1906 was elected, and is composed of representatives of the medical profession and of missionary societies, nurses, and students. Since the foundation of the Union two years ago, meetings have been held in 34 London hospitals and infirmaries, and in the provinces, and nearly 300 nurses have joined as members and associates; of these 10 have already gone abroad. A copy of the report and all information concerning the Union can be obtained from the Secretary, Miss Kathleen Miller, 73 Mildmay Park, N.

### A STRANGE PATIENT.

A strange patient is now under treatment in one of our East End hospitals for children. A sister going into the small garden early the other morning discovered a hen, with its leg stretched out and stiff, lying upon its side in the chicken run. It was gasping and thin, and seemed to be suffering from pneumonia. Brought in to the office fire and dosed with warm brandy, it gradually improved, and in two days was cured of its chest trouble; yet it was still unable to stand, and lay helplessly upon its side with no power to feed itself. Then sister begged medical advice, and a consultation of doctors resulted in a verdict of dislocation of the thigh with synovitis; but, alas for the infallibility of the profession! the diagnosis proved incorrect. No treatment was recommended, and sister, unwilling to lose a patient, was at her wits' end. Fortunately the laundress, making a business call, remarked, "I hear you have a hen very ill. I was brought up on a farm and understand all about poultry. May I have a look at it?" After a short inspection, she diagnosed it as a case of paralysis, to which disease she stated hens are prone, brought on by cold. She recommended the sister to rub the feet and legs with warm oil, and if it did not improve in two days to destroy it, as the case would be hopeless; but if at the end of that time it commenced to walk a little, it would probably quite recover. Under this treatment, and with a luxurious diet of water-cress, eggs, oyster shell, and apples, it began to improve, and is now convalescent, though it still remains a patient in sister's office, knows her voice when she calls, and roosts at night under her table. On cold days it well earned the name given it of "Alice-sit-by-the-fire," and it is to be hoped will repay all the care bestowed on it by laying many eggs in the future for its little fellow-patients in the Children's Hospital.

### SHORT ITEMS.

Miss K. Roscoe has been appointed provisionally staff nurse in Queen Alexandra's Imperial Military Nursing Service.—At the quarterly meeting of the Governors of the Middlesex Hospital, Nurse Caroline Drake was granted a pension of £17 6s. 8d. on her retirement from the Trained Nurses' Institute after 20 years' service in the hospital.—The annual meeting and conversazione of the Royal British Nurses' Association will be held in the gardens of the Royal Botanic Society of London, Regent's Park, on Wednesday next. Admission to the gardens will be at 2 p.m., and the meeting will be held in the Museum at 4.—Miss Mary Luard, who died on Tuesday at the age of 80, at Witham, Essex, organised a small band of women nurses when the district some years ago suffered from an epidemic of typhoid fever, personally superintended their work, and subsequently founded a diocesan home for trained nurses.

### The Nursing Outlook.

"From magnanimity, all fear above;
From nobler recompense, above applause,
Which owes to man's short outlook all its charm."

### THE ADVANCE IN NURSE TRAINING.

The new edition of "How to Become a Nurse: The Nursing Profession; How and Where to Train," should be full of interest to hospital and nurse-training authorities, as well as to every nurse who takes an interest in the profession. It contains accurate information, up to date, concerning the requirements of each training school and hospital where probationers are received, and it reveals the enormous advance in training which has taken place, in the last three years, especially, all along the line. Taking, for instance, hospitals and infirmaries with 100 beds and upwards where nurses are trained, it will be found that, with the exception of the London Hospital, the Salisbury and Worcester Infirmaries in England, the Perth Royal Infirmary in Scotland, and the Jervis Street Hospital in Dublin, every one of these institutions now requires at least three years' training before granting a certificate. A fact like this eloquently testifies to the advance made by the great training schools, whilst it exhibits the force of public opinion in influencing nursing matters in these days. Few things could be more encouraging. These facts too must prove helpful to the Parliamentary Committee now sitting on Nurse Registration, by showing them the way to effectually handle and settle the questions upon which they have been receiving evidence during the last two sessions.

This book further affords ample evidence of the strength and weakness of nurse-training in the United Kingdom in the present day. Its strength is revealed by the facts which we have just quoted, and its weakness may be proved by an inquiry into the increase in the number of private adventure homes and agencies for the supply of nurses to the public, and by the fact that there are 195 hospitals and small institutions with 50 beds and under which advertise for probationers and profess to train them, in addition to the large number of fever hospitals and smaller special institutions, which grant certificates to assistant nurses after one or two years' work, in these establishments. It appears that some 500 probationers are annually received into the 195 hospitals and institutions with less than 50 beds. This shows how great is the demand for instruction in nursing in the present day. It reveals, too, the ignorance of the consequences to herself which a nurse may suffer by entering a hospital as a probationer which is too small to enable her to be fully trained or to obtain a certificate which will pass muster everywhere. We hope that the warning given by the Editor in "How to Become a Nurse" on page iv. of the new edition will be taken to heart by all whom it may concern. The Editor points out, that probationers and their friends do not sufficiently realise, that it is far better to wait for a vacancy in a recognised training school, the certificate of which will give the nurse a definite standing, than to take a so-called probationership in a very small institution which cannot afford her full facilities to complete her training in a satisfactory manner. To succeed in her profession every nurse must first obtain adequate experience and training, and possess a certificate which will pass muster, wherever she may present herself, as a candidate for work.

"How to Become a Nurse" further contains complete figures of the yearly average vacancies and the yearly applications for training at 500 hospitals, infirmaries, and kindred institutions. At the larger hospitals the yearly applications are so numerous that the average vacancies in the nurse-training schools are only equal to about one-fifth of the number who seek to be trained. Of course, the 36,000 applicants each year ought probably to be reduced to one-half by eliminating all who would be found unsuitable for various reasons, and also because it is highly probable that an intending probationer may apply to several institutions in the same year. But assuming that this view be correct, it still leaves us face to face with the fact that only some of the suitable applicants are able, under existing arrangements, to obtain the training which they desire. It is not, of course, possible, to state with certainty, how far and to what extent the increase in the demand for nurses may justify the granting of much greater facilities for training, than are at present available. The important point to remember is, that the supply of probationers is abundant, and that if the public demand for the finished article is not at present met, then steps can readily be taken to develop the training-schools to the necessary extent so as to meet adequately the requirements of the public. At some of the great hospitals every nurse, on gaining her certificate, is offered the opportunity of becoming a member of its private staff, and is guaranteed that she will receive the larger portion of her earnings in that capacity. If the Parliamentary Committee determine to recommend that the supply of pseudo-nurses, who unjustly compete at present with the certificated nurses, shall be restrained and put an end to, by bringing the private adventure homes and agencies under inspection and control, the demand for really efficient nurses must largely increase. For these reasons, and others, it is highly desirable that the authorities of the great nurse-training schools, and all who have the interests of nurses at heart, should carefully consider the problem here presented, with a view to its prompt solution and the removal of any existing abuses.

### The Nursing of Sick Children.

By James Burnet, M.A., M.B., M.R.C.P.Edin., Registrar, Royal Hospital for Sick Children; Clinical Tutor, Extramural Wards, Royal Infirmary; and Physician to the Marshall Street Dispensary, Edinburgh.

### IX.—DISEASES OF THE NERVOUS SYSTEM (*continued from p.* 141).

A very common nervous disorder met with in children is that known as night-terrors. The child wakes up after a more or less sound sleep, and usually sits up in bed staring wildly round him. He cannot understand where he is, and often fails to recognise his mother or nurse. This condition is certainly much more common in nervous children, though it is sometimes met with in those who have hitherto shown no evidence whatever of nervousness. Thus we find it in children who are suffering from some digestive disturbance, constipation being in many cases a predominant factor in the causation of night-terrors. It is also fairly common in rheumatic children, and in those who are affected with St. Vitus' Dance.

When night-terrors are present the nurse should try to pacify the child, who will at first appear to ignore everything she says. After a time, however, if she talks quietly to him he will usually recover and recognise that after all he is in bed with nothing to fear. Very often by giving the child a warm drink the attack may be cut short, as this presents something real to the mind, instead of the fancied vision which has given rise to the sudden fit of terror. In order to avert repeated attacks the nurse should see that the child is not over-excited during the day, and that he is not allowed to run about just before going to bed. Sometimes a tepid sponge bath just before retiring to rest has a very beneficial effect, and so also has cold douching of the spine in the morning. Medicines may also be given with a view to soothing the over-sensitive nervous system of the patient, but the nurse must never on any account administer these without express orders to this effect from the physician.

Bed-wetting is such a common disease in children of both sexes that we think it deserves to be considered amongst nervous disorders. This is undoubtedly a most troublesome condition, causing the nurse much annoyance. The management of these cases requires a good deal of care on the part of the nurse, as, unless the child is properly taken in hand, no amount of medicine will do him any good. Bed-wetting, or nocturnal incontinence of urine, is merely a continuance of what is the normal infant state. It may result from irritation due to worms, constipation, or a long foreskin, all of which conditions must be rectified. On the other hand, it may result from general debility, and, in the majority of cases, tonic treatment is of first importance. Cold sponging, and especially douching of the spine, is frequently found to be very beneficial. Otherwise the diet must receive attention. Such children should be kept largely on a diet of fish and milk, including milk-puddings, with perhaps the occasional addition of eggs and fruit. No fluid whatever should be given after 6 p.m., and the child should be put to bed at 7 p.m. Before retiring for the night he should be made to empty the bladder, and at 9 p.m. he should be waked up for this purpose again. He should be made to sleep on a hard mattress, and under the blanket or sheet should be placed a mackintosh, while under the child himself the nurse should put a layer of absorbent cotton-wool. These precautionary measures will certainly prevent the uncomfortable results arising from the bedding getting wet through, as would otherwise be the case. In male children nurses are very apt to tie a tape round the penis in order to prevent the escape of urine; but this measure cannot be too strongly condemned, though we have reason to believe that it is sometimes resorted to by those who ought to know better.

We now come to consider the very important nervous disease known as St. Vitus' Dance, or, as it is scientifically termed, chorea. This disease is very often simply a manifestation of rheumatism, though it may be associated with the presence of worms in the intestinal canal. When a child is affected with chorea he throws his limbs about in a most erratic fashion. They go through all sorts of wriggling movements, while the hands are twisted aimlessly about. The shoulders are continually shrugged, and the face exhibits movements of contortion, while the tongue, if protruded, is jerked back in a rapid, spasmodic fashion.

Children suffering from chorea should always be confined to bed. They must be kept by themselves in a room which is as far removed from noise as possible. The nurse should see that the bed be so prepared that the child cannot possibly hurt himself during his vigorous movements. In severe cases it may be necessary to take special precautions lest the child wriggle himself out of bed altogether. It is often advisable to have the bedding and pillows made of the softest possible material in bad cases, as when the movements are at all violent the child is apt to produce sores, especially over the back, as well as about the head and face.

Massaging the limbs is often found to aid in bringing about recovery in these cases. Otherwise the nurse should not attempt to stop the movements. Any such attempt will be sure to meet with failure, as the more the patient is taken notice of by the nurse the worse will his movements become. As regards diet, nothing should be allowed save milk and milk puddings. In every case the bowels and bladder should receive special attention, as we often find that intestinal and urinary difficulties present themselves if the nurse neglects these important matters. Arsenic is often ordered by the attending physician, and that, too, in somewhat large doses. When this produces diarrhœa, pains in the abdomen, or sore eyes, the fact should be immediately reported by the nurse. A careful record of the pulse and temperature should be kept in every case, and a note should especially be made of any rapidity or irregularity in the former, and any sudden rise in the latter. The reason for this is that heart complications of a serious nature are not uncommon in the course of this disease. A change in the character of the pulse, or a sudden rise of temperature, may herald the commencement of heart mischief, and by noting these the nurse may be able to materially aid the physician in recognising the onset of such complications.

The feeding of serious cases of chorea often presents insuperable difficulty. When the movements are very violent it may be quite impossible to give the child any nourishment whatever by the mouth. As a rule, in such cases rectal feeding becomes absolutely necessary, but it should never be resorted to before mouth feeding has been given a genuinely fair trial. The reason for this is obvious. Children suffering from severe attacks of chorea show signs of exhaustion very rapidly, and unless they are fed often and sufficiently they may die from simple inanition. It is well, therefore, in cases presenting difficulties in the way of mouth feeding to give a little often rather than attempt to get the child to swallow a large quantity at any one time. Sometimes we have found a feeding cup with a rubber tube attached to the mouthpiece simply invaluable. This rubber tube can be placed far back in the mouth, and, as a rule, the food can be got over in this way, especially if milk only is given, and that in small quantities.

Before leaving the subject of nervous diseases, we would warn nurses that in all the cases we have been considering the feeding and bodily cleanliness of the patient must occupy a foremost place. Neglect of these may lessen very materially the chances of the child's ultimate recovery. Medicines, too, when ordered, should be administered at the proper intervals, and the strictest attention be paid to the instructions given by the physician in attendance. The patience of the nurse will often be greatly tried in these cases, but her reward will be comparably great if the patient recovers, and should a less fortunate result be met with she will at least feel that she has done her duty.

### The Nurses' Clinic.

### THE DISPENSARY. BY A CERTIFICATED DISPENSER.

### PILLS.

Pills should be made perfectly round and not too large, never containing more than five grains each if it can be avoided, unless the ingredients be exceptionally heavy—as bismuth, calomel, etc. It is best to have a very shallow, thick, wedgwood mortar, with a small but long pestle, as the mass, instead of being worked round and round the mortar or pounded, requires to be firmly kneaded; a certain amount of force is necessary, and the friction softens the mass, so that less excipient will be wanted to produce a workable pill. The first thing to do, after having read over the prescription, and decided what excipient to use, is to weigh out the ingredients, commencing with the ones that require powdering, and after all the dry powders have been mixed the soft extracts are added, and the mass worked up in a mortar. It should be explained that the "excipient" is some soft substance, as gum or mucilage, syrup, or extract of gentian, etc., with which it is necessary to bind the powders together, the choice of which is often left to the discretion of the dispenser. Besides those mentioned above there are many others, different ones being suitable to various ingredients ordered. After all has been weighed out and worked into a fairly stiff mass in the mortar, it is a good plan to place it in the palm of the hand and knead it well with the fingers for a few minutes to soften and toughen it, it is next put on to the marble slab of the machine and rolled out with the flat side of the handle, in much the same way as pastry, taking care, however, not to roll it flat but to keep it round and the same thickness throughout; if it tapers it makes the end pills smaller than the middle ones when they are cut; the slab should be powdered with French chalk or carbonate of magnesia to prevent the mass sticking to the slab. After having rolled it out satisfactorily, it is picked up in the fingers or rolled on to the grooved end of the machine, and with the back of the handle pressed firmly down, and by a series of rapid short movements the pills are cut into equal sizes; they will then generally require rounding off perfectly, either by being again placed in the palm of the hand, dusted with the French chalk or magnesia, and lightly rotated with the tips of the fingers, or they may be replaced on the slab, and the same process be performed with the pill-finisher. Various substances are employed for coating pills, usually either French chalk or talc, either alone or sweetened with sugar; sometimes only the latter is used. Gelatine is frequently employed; silver leaf used to be a favourite covering until the introduction of chalk or "pearl-coating." Whatever be employed the pills should be allowed to harden before it is applied, otherwise discoloration occurs either from reaction between the ingredients and the silver leaf, or else from the moisture saturating the white coating.

For silvering or gilding, the pills having been made sticky by a few drops of mucilage of acacia, are put into a covered pot containing gold or silver leaf and rotated quickly for a few minutes, after which the pills are put into a clean pot and again rotated to burnish them. In pearl coating the pills must be free from powder and first well varnished; when the varnish is dry they must be moistened again with a mixture of mucilage of tragacanth and half a drachm to the ounce of simple syrup, rotated in the usual way in a covered pot containing very finely powdered French chalk, and burnished in a dry clean pot.

The following are a few of the principal excipients in general use:—

Salts easily soluble in water require very careful addition of an aqueous excipient; glycerine should be avoided. Spirit should not be used when there is much resin in the pill. Water is used with substances which themselves contain a binding material as gum or soap; three minims of water suffices for 12 five-grain pills.

Scale preparations can also be made with water, but it tends to make them hard. It is also a good excipient for opium pills; its real use is to diminish the bulk.

White beeswax is used with the colourless oils.

Castor oil is excellent for making camphor pills.

Oils should be made into pills usually with soap and pulv. trag. co.

Copaiba should be mixed with calcined magnesia, 16 parts to 1 of magnesia, and set aside to solidify.

Creosote should have half a grain of curd soap, and liquorice powder in a sufficient quantity to each drop. The same applies to croton oil.

Pills containing a fixed oil and beeswax are best made by dissolving the beeswax in an evaporating dish, the oil being stirred in, and they are rolled out when sufficiently firm.

Soap should not be used for salts of lead, iron, bismuth, copper, or mercury, as oleates would be formed. Glycerine of tragacanth is good for ingredients not easily soluble in water, as camphor and menthol, which should each be

THE NURSES' CLINIC—*Continued.*

rubbed up separately with a little pulv. tragacanth; if rubbed together they liquefy. A white excipient should, if possible, be used for white powders. Hardened pill masses can be rendered more workable by rolling on a warmed slab. Digitalis leaves must be finely powdered and rubbed up with extract of gentian.

Essential oils should not be used with wax unless absolutely necessary, on account of its insolubility and high melting point.

Extracts as cascara, cannabis indica, etc., should be mixed with some powder, as pulv. tragacanth or liquorice powder. When Ferri sulphas is ordered, always use the exsiccata—two and a half grains of the latter to every four grains ordered.

Pepsin requires to be made very quickly, with half its weight of dilute glycerine. Pix liquida (tar) must be solidified with an inert powder, as flour or liquorice powder, made very hard and rolled on a warmed slab.

In making pills of potas. permang. organic substances must be avoided, or decomposition will take place, owing to oxidation; it also decomposes most inorganic substances. Kaolin ointment (composed of kaolin and vaseline) is one of the best excipients for permanganate of potash. Confection of roses makes an excellent excipient for sulphate of quinine pills. If small pills are required a little dilute sulphuric acid may be added, or an eighth of its weight of tartaric acid; care, however, must be taken not to put more tartaric acid, as it acts chemically on the quinine and would form tartrate of quinine instead of sulphate. Quinine hydrochloras can be reduced with a little hydrochloric acid. Soft extracts which are not hygroscopic should be evaporated to a suitable consistence. Hygroscopic extracts must be hardened with an inert powder.

When glycerine is used as an excipient it should be mixed with an equal quantity of water, as it absorbs moisture, causing the pills to become soft.

Physicians often order pills to be made of perhaps an eighth or a fifth of a grain of some substance, with no other directions as to what is to be made up with it, and as it stands to reason that a pill of the size of an eighth of a grain cannot be sent to a patient (who would hardly be able to see it!) the dispenser must add some powder to increase its bulk to a grain or two, sac. lac. or pulv. tragac. co. being most commonly used. Other doctors, again, have a way of ordering a fraction of one ingredient, as one-sixteenth of a grain in each pill, and 30 to be sent, in which case the best plan is to make 32 pills, to avoid the fraction and throw the extra two away.

### Select Committee of the House of Commons on Nursing.

### SIR HENRY BURDETT PROPOUNDS A COMPLETE SCHEME.

The Select Committee of the House of Commons met on Thursday last week.

### Evidence of Dr. Greves.

The first witness was Dr. Greves, of Bournemouth. He said his 25 years' experience as a medical man had convinced him of the necessity of State registration of nurses as a guarantee that they had had proper training. He thought that from two to three guineas ought to be sufficient for the expenses, though perhaps that might not cover the cost of examination. Registration would protect the medical profession, and, therefore, the public, from incompetent nurses, and would also improve the status of nurses by bringing them all up to a certain standard. He did not think there was any fear of trained nurses in country districts taking cases from the medical men. He said at first that he thought there would be room for a certain number of nurses not of the highest training in country districts in cases where no great skill or experience was required; he would not prohibit them or prosecute unless they called themselves registered nurses; but on being pressed by Dr. Hutchinson he expressed himself in favour of compulsory registration of all nurses, and making it penal for anyone else to nurse for gain. He thought the added cost for the extra training would be obtainable from the same charitable sources as now, and that there would be no difficulty in getting a highly trained nurse to do the work necessary to be done when nursing in cottages and in villages. What was required was protection from bad nursing.

### Evidence of Sir Henry Burdett.

Sir Henry Burdett, in reply to questions by the Chairman, said he had given a great deal of work and thought to the training of nurses, and had carefully followed the evidence given before that Committee. He spoke not as being now engaged in or connected directly with hospital or nursing work in a particular institution, but as having a great deal to do with nurses through the Pension Fund and as one who was consulted by, and frequently discussed the subject with, all sorts of people connected with such work.

He would first like to deal with the question of numbers which had a most important bearing on the whole question. He thought he could show that the number of people affected by this inquiry had been greatly overstated. The census returns under the heading of "sick nurse," "invalid attendant"—gave 64,214 females for England and Wales, 7,812 for Scotland, and 2,818 for Ireland—in round figures a total of 75,000. From these must be deducted about 9,000, who were either too old or too young to be trained nurses, leaving 66,000 according to the last census. But he had ascertained from the Registrar-General that the term sick nurse included aspirants Sisters of Mercy, who were really not trained, with the exception of a few at the Hospital of SS. John and Elizabeth, hospital sisters, and monthly nurses—and that must mean many thousands—nurses, tender, *i.e.* those in charge of old people and so on. In fact, so-called nurses, whether trained, wet, hospital, or lunatic attendants (private houses) all went down as nurses. After the most careful consideration and inquiry he had formed the conclusion that some two-thirds of the 66,000 could not be regarded as trained nurses for the purposes of that inquiry, and that the right number for the Committee to consider was from 22,000 to 25,000.

Arriving at the figures in another way, there were 12,727 nurses employed in the 499 institutions—general hospitals, Poor-law infirmaries, special hospitals with 50 beds and upwards, and in nursing institutions managed by a committee, and Government departments, where nurses were employed but not trained, and 678 in the 195 institutions having less than 50 beds—a total of 13,405 working in the institutions to-day. Then, calculating from the number of those undergoing training, it must be assumed that there were now another 14,000 who had been certificated during the last 10 years, so that in all probability the total number of trained nurses working in the United Kingdom did not exceed 30,000 if it even touched 25,000. The difference between that figure and the very much larger number that had been talked of was made up of the pseudo-nurse, of little or o experience, save at a fever, special, or cottage hospital, and the "born nurse." Nurses reached the public from the hospitals, the nursing institutions, and the co-operations under a committee, the private adventure homes and private adventure co-operations without a committee, and the private nurses working on their own account. It was the large profits made by private adventure homes which were the main cause of the present evils of supply, since they made the pseudo-nurse possible, and the greater the demand the more variegated would be the supply. These private adventure homes and agencies should all be registered and inspected. They paid nurses £30 to £35 a year, and the nurses' earnings in private work were about £96 in London and about £70 in the country. He thought the nurses should be protected. It was not necessary to deduct more than 7½ per cent. from their earnings for working expenses of all kinds; indeed, the Nurses' Co-operation now deducted only 5 per cent. from many nurses' takings. Having been one of those instrumental in founding the Nurses' co-operation, he knew how large must be the profits of the private homes and agencies, and how great the temptation for them, when the demand was keenest, to supply untrained nurses.

As to the training of nurses, speaking generally, the position was satisfactory. The period of training differed somewhat, but it was now the almost universal practice to require a three years' course; where the certificate was withheld till the end of the fourth year that was a matter of contract between the probationer and the hospital. At the best hospitals preliminary training was given before admission to the training school. Training in the wards was under the supervision of the sisters, and supplemented by lectures by the matron or her assistants and by members of the medical staff. The first year was usually devoted to preliminary instruction, and the last two to practical courses in children's and special departments, and sometimes to private nursing. He thought it would be well to have a practically uniform curriculum and certificates. It was an anomaly that special and fever hospitals should grant any but special certificates, for they could not give instruction in all branches of work. The real training of a nurse came, of course, from experience by observation. The technical part of her work could be generally mastered in 12 months, the rest was largely experience. Hospitals should not give probationers an undue proportion of linen-room and housework, nor keep them too long in one ward without seeing special work. No probationer should be sent out to private nursing till she had completed her training. These were all points reforms in which he hoped would result from that inquiry, and also co-operation among those engaged in training nurses. All classes of the nation, he thought, owed gratitude to the nurse-training schools and the hospitals, which had brought nursing to a high state of efficiency.

The advocates of State registration claimed that it would secure a higher average of efficiency; protection against the pseudo-nurse; protection for the public and the medical profession against drunken, dishonest, and immoral nurses by giving power to cancel the registration certificate; and uniformity of training and curriculum. He proposed to show how these objects could be gained by better, simpler, and less expensive means. According to the evidence of the chief promoters of it, the passing of the Registration Bill would result in the letting loose on the public of some 30,000 women who were not properly trained, but were in practice at the time. The Bills before Parliament would not give direct representation to the existing schools and hospitals which were now training and certifying nurses. A fee of five guineas was to be demanded, though doctors only paid £5, a fee that he considered prohibitive. He failed to see also why a nurse alone of all men and women in professions should be required to be re-examined after taking her diploma. If she did not pass she suffered the humiliation and the loss of being cut off from her profession, at the end of three years' hard work, though she might have obtained a certificate, with honours, of technical efficiency and personal character granted after examination by her training school.

The 60,000 women waiting to register were to contribute £120,000 to place the Registration Board on a firm basis, and the certificated nurses who afterwards submitted themselves for re-examination were to contribute the £5,000 a year estimated to be required for the payment of the 21 or 32 members of the Board under one or other of the Bills. After criticising the composition of the proposed Board, Sir Henry continued: "It is, however, I think, possible by registration to remedy the defects in the present system. This is my proposal. Let Parliament enact that all training-schools for nurses and all hospitals and institutions that train nurses in accordance with a given standard shall be registered and inspected. Make the registration of each such establishment conditional on the training of nurses being carried out in such a way as to fulfil the standard laid down; conditional, too, on examinations being held, on their due inspection on some such system as that which governs the proceedings of the General Medical Council, on the granting of certificates in a certain form, on the publication of a register each year of all nurses trained and certificated by each such school and hospital, on the post-graduate training of all certificated nurses who desire it by the school where they were trained; provide further that each training school and each hospital which trains nurses shall afford its nurse-graduates the opportunity if they wish—(1) of joining the private nursing staff of the institution; and (2) of having secured to them in liberal measure the payments received for their work, as is already done at Guy's. In this way the nurses would come to look to the school where they were trained, as the best nurses do to-day, and the school authorities would be enabled to keep in close touch with all their graduates from year to year to the benefit of the profession and the public. Let the Registration Act grant powers to each registered training school and hospital to cancel certificates and strike a nurse's name off the register for infamous conduct in a professional sense after due inquiry, with the right to the nurse to appeal to the General Nursing Council to be set up by the Act. This Nursing Council should consist of representatives of the nurse-training schools and hospitals which train nurses, and of five elected members representative of the nurses registered under this scheme—three for England and Wales, one for Scotland, and one for Ireland. I believe that an Act of this kind would prove efficacious and highly popular with all concerned. The financial arrangements would be simplified by the fact that the registration fees would be paid by the institutions and not by the nurses, who really, for the most part, cannot afford them. I would further propose that all nursing homes and all agencies and institutions supplying nurses to the public should be registered and inspected. Registration should be conditional upon the supply of nurses to the public through these agencies being confined to efficient and qualified women who had attained a certain standard and were registered accordingly. This would cut at the root of existing evils, and within a very few years they would probably be entirely removed.

"One other matter to be considered is that it is essential that provision should be made for the floating army of irregulars—nurses who have not had the advantage of three years' continuous training in a single hospital, but who may possess excellent technical and moral qualifications, and may in fact be useful and satisfactory nurses. I think myself Lord Rothschild's scheme for the higher education and training of nurses might usefully undertake the work of organising examinations for the benefit of this army of irregulars. Those members who could pass the test could be granted a certificate which would entitle them to rank as qualified nurses. Something of the kind must be done in justice to a large number of worthy women, many of whom are most useful in the day of sickness and who could show that their knowledge and experience entitled them to the certificate which that Society could thus secure them."

Proceeding, Sir Henry explained that in the United States registration was made possible by the much greater co-operation between those who trained nurses. There they had first established a Society of Superintendents of Training Schools representing all the best opinion in the States. They were thus more co-ordinated, and that had prepared the way for registration. In New Zealand the hospitals were under State control; and in Australia they were subsidised by the Government, and so registration was in reality compulsory. Although the majority of nurse-training schools were opposed to registration, as proposed in the Bills before Parliament, there was a movement among them in favour of something being done, and he thought they ought to come together and formulate proposals. The scheme he had put forward for the registration of training schools would get the public to look to the hospitals, and so give the best guarantee of efficiency in nursing, which could only come from efficient training. Every properly constituted training school at present kept a register which gave a record of training and of subsequent service. Training had taken a tremendous leap forward in the twelve years since he gave evidence before the House of Lords. The credit for the fact that three years was now the recognised period must be given to Mrs. Bedford Fenwick, who had done excellent service in that matter.

Replying to questions by the chairman Sir Henry emphasised the importance he attached to uniformity of curriculum, not of training. By the scheme he had propounded for the registration of all training schools they would secure the co-operation that was so much wanted. There would be a Central Nursing Council, following the lines of the Medical Council. The Nursing Council would represent both the schools and the nurses in the same way that the Medical Council represented the bodies which granted the diplomas and the doctors as well. It was essential that the examinations should be conducted by the leaders of the schools in charge of nurse training. He had consulted a good many such, and hoped his view would be welcomed by the nurse-training schools generally. They were keen on something being done, for it was clear they could not go on letting the pseudo-nurse go out to the danger of the public, but must take steps to bring about an increased supply of efficiently-trained nurses. What the doctors wanted was some method of sifting the nurses and preventing all sorts of inefficient people being sent to patients. His scheme would do that and also keep them up to date by the post-graduate course. There would be a central register, independent examiners; the school would examine and grant certificates as now, and would then send forward a recommendation to the Nursing Council that the nurse was qualified to be registered—exactly the same as the Medical Council with the training school in place of the University. The Nursing Council would supervise by sending visitors to the examinations just as the Medical Council did. They had in the Medical Council a model to go on which had been tested by years of experience, and was working satisfactorily. The proposal to pay the members of the Registration Board under the bills £5,000 a year was in startling contrast with the fees paid to members of these Boards in the United States, where each member received from one to five dollars for each day's attendance. The number of members on these American Boards was fixed at five members.

At this point the Committee adjourned.

### Evidence of Mr. J. R. Motion.

The first witness on Tuesday morning was Mr. James Russell Motion, Inspector of the Poor and Clerk to the Parish Council of Glasgow. He said that he gave evidence at the instruction of his Council, who had passed without discussion a resolution to the effect that they were in favour of State registration of nurses, and were willing to supply any information on the subject which the Committee might desire. Mr. Motion explained that, in consequence of a sumof £20,000 available under a Treasury Minute by the Local Government Board in Scotland for medical relief, it was the practice to train nurses for Poor-law work, and the salary and maintenance of the nurses after training was found out of this fund. The Local Government Board was able, therefore, to insist on all nurses in its employ being registered, registration meaning two years' training at a public institution and the passing of an examination. No fee was charged to the nurse. The Parish Council of Glasgow had attempted to institute a scheme, whereby if a nurse trained for a third year and during a fourth year took up mental nursing, she should have a higher salary. This attempt, however, had been unsuccessful, chiefly through the feeling among general hospital nurses that mental nurses were not equal to them and their consequent dislike to mix with them.

Mr. Motion had asked four matrons in large institutions in Glasgow their opinion as to the fee which should be demanded from nurses for State registration, and they had replied that half a guinea would be reasonable. His Council employed 227 nurses, of whom 144 were probationers. They employed no nurses (after a three-months' probation) who were not either registered or preparing to be registered. He thought that there should be some means whereby a nurse should have a parchment certificate, showing her work for the last five or ten years, as the case might be; the doctor at one or two of her cases during each year might endorse this certificate in some way.

Mr. Motion explained that there was no uniformity in their examination for registration, each medical officer setting his own examination. The examination was oral and written, but not, he believed, clinical. There was no specific guarantee of character, though they would not accept a nurse for examination without inquiry. If a fault was proved against her after registration she was struck off the register and not employed by any of the local authorities; but, of course, she was free to practise independently. If State registration were introduced it would be, of course, unnecessary to continue their present system. He felt sure that there would be no shortage of nurses in Glasgow if the proposed registration, after three years' training, were introduced. He was unable, however, to give any very definite figures as to the present supply, his work having been confined to Poor-law institutions. His Council had not really considered whether registration should be compulsory or voluntary, but he thought that if compulsion were necessary, so that it should be effective, they would be in favour of compulsion.

### Evidence of Miss Shannon.

Miss E. Shannon, Matron of the Western Infirmary, Glasgow, then gave evidence. She said that there were 150 nurses in the hospital, which had 430 beds, of whom about 40 were first year probationers, 30 second year, 36 third year, and 40 fourth year. The nurse signed a contract with the hospital after three months' trial to stay at the hospital four years, though she received her certificate at the end of the third year. She did not anticipate any shortage of nurses if a three years' registration scheme were introduced. She had about 600 applications in the year which she was unable to accept. She admitted that she had only considered the question of registration from the infirmary standpoint, and not looked at the difficulties which might accrue in rural districts. She thought that five guineas would be a reasonable amount to ask the nurses to pay, despite the fact that most of the nurses in her hospital were the daughters of farmers and tradespeople, and very few from the "gentle" class. She was of opinion that a voluntary system of registration would be sufficient.

EVIDENCE OF MR. DUNCAN

The last witness was Mr. G. W. Duncan, Secretary to the Central Midwives Board. He expressed no opinion on the question of registration, but merely described the operations of the Board. As the two years' grace period had only just been completed, it was too early, he said, for him to be able to give any account of expenditure by the Board under its permanent conditions. At present 22,000 women had received certificates, without examination, as midwives at a charge of 10s. The profit made by the Board was about £7,500 so far, but they anticipated a large increase in expenditure as soon as the normal system was at work, and they would, after this capital was expended, have to call on the County Councils for the deficit. The fee in future for examination and registration was one guinea, and he did not think it desirable to raise the fee. Of this 7s. would be paid to each of the examiners, who examined each examinee, so that there was only 7s. left for all other expenditure.

He mentioned that they had struck off three names from the register since January, and there had been no appeal against the Board. They anticipated some difficulty in respect of evidence, as they had no power given them to compel witnesses to appear. The Board had power to license institutions for the training of midwives. They had found no difficulty in getting information from the institution, which had to fill up certain forms of inquiry; the medical officer of health also supplied information. They had had to refuse licenses, not for alleged scandals, but for unsuitability and non-compliance with the requirements laid down.

### Society for the State Registration of Trained Nurses.

THE third annual meeting of the Society for the State Registration of Trained Nurses was held at the Medical Society's rooms, Chandos Street, last Friday afternoon. The meeting occupied less than an hour, the chief business being the reading and adoption of the report and the election of the members of the executive committee, with a short address by Mr. R. C. Munro-Ferguson, M.P., on Parliamentary procedure in relation to the passage of Bills into law, with special reference to the Society's Bill for the registration of nurses, which this Session was introduced by him.

In the absence of the president, Miss Stevenson, who sent a letter regretting her inability to attend, the chair was taken by Miss Isla Stewart, matron of St. Bartholomew's Hospital. It appears from the report that since the last annual meeting 300 applications for membership of the Society from matrons and certificated nurses have been accepted, making a total of 1,505 since the formation of the Society three years ago.

The report gives a synopsis of he work accomplished, and of the various stages of the movement for registration. After referring to the appointment of the Select Committee both last Session and this, and to the constitution of that Committee, it sets out the names of those who have given or are to give evidence before the Committee.

The protest of the Society against the petition to the Board of Trade for the incorporation c a society for promoting the higher education and training of nurses is also given in full in the report.

The Society has decided to affiliate itself to the International Council of Nurses.

Miss Stewart referred to the lay association for promoting the State registration of nurses, which had recently been formed, and was largely an outcome of the society.

### Central Midwives Board.

SOME PENAL CHARGES.

A SPECIAL meeting of the Central Midwives Board was held at the Board-room on Thursday last week for the consideration of three penal cases. There were present Dr. Champneys (in the chair), Dr. Dakin, Mrs. Latter, Miss R. Paget, Miss Wilson, and Mr. Parker Young.

The Board heard the evidence against Annie Bord, a certified midwife, of Aldershot, and examined her. They found the charges of not having sent for medical help and of having disobeyed the rules with regard to disinfection in connection with a fatal case of puerperal fever occurring in her practice to be proved, and ordered her certificate to be cancelled. The Chairman, in stating the Board's decision, said that midwives should take warning that, with the lives of women in the balance, ignorance of the rules could not be accepted as an excuse.

The charges against Georgina Eliza Price, a certified midwife, formerly in the employ of the British Lying-in Hospital, were of malpractice and drunkenness. Mrs. Price was represented by her solicitor and counsel. After considerable deliberation on the evidence before them the Board came to the conclusion that the charges were not sufficiently proved to order the certificate to be cancelled, but the chairman severely cautioned Mrs. Price as to her future behaviour, warning her that another time she would not escape so easily. He expressed the thanks of the Board to Nurse Girdlestone, who had come up from the country as a witness against Mrs. Price, for her public spirit, and for giving her evidence well under trying circumstances.

In the third case, that of Martha Maria Herbert, a certified midwife of Manchester, who did not appear, the Board ordered her to be severely cautioned with regard to various infringements of the rules, and warned that a note would be made against her name.

The following midwives were approved for the purposes of signing Forms 3 and 4:—I. Beard, A. C. Chowne, A. Franklin, A. M. Palmer, E. Beckwith, M. Blow, R. Fuller, A. Gosling, H. Macdonald, Eliz. MacKenzie, A. K. Owens, M. J. Barrett, R. Bush, K. M. Greetham, L. M. Lee, Annie Michie, E. M. Neison, E. M. Sleight.

### To Nurses.

WE invite contributions from any of our readers, and shall be glad to pay for "Notes on News from the Nursing World," or for articles describing nursing experiences at home or abroad dealing with any nursing question from an original point of view according to length. The minimum payment is 5s. Contributions on topical subjects are specially welcome. Notices of appointments, letters, entertainments, presentations, and deaths are not paid for, but we are always glad to receive them. All rejected manuscripts are returned in due course, and all payments for manuscripts used are made as early as possible after the beginning of each quarter.

### Precautions for the Prevention of Splint Sores.

### EXAMINATION QUESTIONS FOR NURSES.

The question was as follows:—In the case of fractured thigh put up in a long splint and extension, and in a case of hip disease treated with a splint and extension, what precautions should you take for the prevention of splint sores?

### First Prize.

In cases of fractured thigh or hip disease, with long splint and extension, the parts most likely to get sore are the hip bone, ankle, and heel, through pressure of splint or bed; knee and ankles through strapping chafing; and space above heel over tendon Achilles, through strapping or bandage cutting.

To prevent all these I should, before bandaging on the splint, place two long pads, not too thick, one reaching from the armpit to waist, and another from just below the knee to above the ankle; these will take all pressure from sides. When bandaging I should begin well above the ankle, this will leave the ankles and the space above heel free. If a foot bandage is considered necessary, put a separate one on after, which can be removed daily and the heel, ankles, and space above the heel properly lathered, spirited, and powdered. This will not interfere with the limb.

To prevent chafing, a piece of boric lint over the knee and ankle before applying extension will keep those parts perfectly smooth.

To raise the heel from a bed do not put the small pad above heel, this only removes the pressure from one part to another, as small ankle pads also do, but put another long pad, fairly wide (or, better still, a smoothly-folded towel), under the leg and splint from below the knee to above the heel. This will keep the heel up and will not put any undue pressure on any spot.

With many years' experience as a surgical sister such cases treated this way have never developed a sore.

The strapping used to fix extension should be two long narrow strips, commenced at top of extension, crossing each other diagonally about three times down the leg. These keep quite firm and do not cut the leg through the constant pulling of the weight as pieces put straight round are apt to do.

"Janet."

### Second Prize.

The long splint should be evenly padded, the pad being made of tow and larger than the splint, all pressure avoided from bony prominences, especially those in the heels and ankles, by placing in between the bone and the splint a circular hollow pad made of cotton-wool lined over with a bandage where the prominent part of bone should rest off the splint. It is no use applying a lump of wool where discomfort is felt by the pressure, but the only thing is to take the splint off and adjust a ring pad where the pain is.

The bandages should not be applied too tightly, as to interfere with circulation, for it may cause swelling, followed by gangrene.

No bandage should be put under the splint and reversing not done on the limb where creases and fold might hurt.

Wedge-shaped pads are very useful when neatly fitted to fill up a hollow space between the limb and the splint.

Finally the foot of the bed is raised up on blocks to prevent the swelling of limb. "Cantharides."

### Defects of the Papers.

The papers are not good this month. Enough intelligent thought has not been given to the subject, with the result that the candidates lay great stress upon a single point, and omit to consider that there are others of equal importance. "Janet" and "Cantharides" win the two prizes, because among a very mediocre collection of papers they are, on the whole, the best. "Janet" takes a more general view of the subject, she therefore takes the first prize. She notices the danger of splint-sore on the outside of the knee, of which danger very few candidates take sufficient heed. Her remarks about raising the heel from the bed are sound. "Cantharides" gains the second prize, because she sees that to cram a piece of wool in when a patient complains of pressure is only to increase that pressure, and double the original evil. The only possible way to avoid pressure-sores from splints is to provide a slight hollow for every bony prominence. I should like to see evidence of more practical common sense in the arrangement of the stirrup for extension; very little is said by the candidates on this, and it is of primary importance. "Rose" alone advises a hole in each side of the stirrup over the ankle-bones—most important and useful—but there are other precautions that might be taken; and I hope when the question is put again that it will produce better answers.

### Honourable Mention.

This is gained by "Rose" and "Fen."

### Question for May.

In nursing a patient with brain disease or injury, from whom it is necessary to keep all light and noise, how would you arrange the lamp and light from the fire at night, and how would you avoid any noise in keeping up the fire?

### Rules.

The competition is open to all. Answers must not exceed 500 words, and must be written on one side of the paper only, without divisions, head lines, or marginal notes. The pseudonym, as well as the proper name and address, must be written on the *same* paper, and *not on a separate sheet.* Papers may be sent in for 15 days only from the day of the publication of the question. All illustrations strictly prohibited. Failure to comply with these rules will disqualify the candidate for competition. Prizes will be awarded for the best two answers. Papers to be sent to "The Editor," with "Examination" written on the left-hand corner of the envelope.

In addition to two prizes honourable mention cards will be awarded to those who have sent in exceptionally good papers.

N.B.—The decision of the Examiner is final, and no correspondence on the subject can be entertained.

Any competitor having gained three prizes within the current year shall be disqualified from taking another until 12 months shall have expired since the first prize was gained.

### Everybody's Opinion.

### HOSPITAL LETTERS.

Miss Young writes from Bamburgh Vicarage, Norwich: Can you, through The Hospital, let its readers know that I have a few hospital letters to give away; I have more than we require just now for our own parish, and some parish not so well off for subscribers to these charities as we are may be thankful of the help. I give a list of the letters I have to spare: 3 indoor for the Jenny Lind Infirmary for Sick Children; 12 outdoor ditto; 2 Children's Convalescent Home at Yarmouth (girls age 3—13, boys age 3—10); 4 outdoor Norfolk and Norwich General Hospital.

### THE ROYAL NATIONAL PENSION FUND FOR NURSES.

Nurse Greenwood writes from 232 Richmond Road, Hackney, N.E.: Will you please spare space in The Hospital for a few words in order to relate my personal experience of the Royal National Pension Fund for Nurses. Eleven years ago my father bought me two pensions in this excellent Fund, one was "returnable," and £6 14s. was paid yearly on my behalf. Since my father's death, three years ago, I have been able to keep up the premiums until this year, when circumstances made it necessary for me to ask for the surrender value of this pension. I explained matters to Mr. Dick, and without any trouble whatever a cheque for £86 3s. 7d. was sent to me last night; during the 11 years only £73 14s. had been paid on my behalf. I cannot speak too highly of the extreme kindness and courtesy I have always met with from Mr. Dick, the secretary of the Fund, Mr. Nairne, the solicitor, and Dr. Potter, the medical referee, whose several aid I have claimed at different times, as being a member of the Pension Fund, and which has never been refused. I did not know any of these gentlemen before joining the Fund, and little did my father think, when he bought the pensions for me, that he had also provided me with three of the kindest and most sympathetic friends a nurse ever had! I should be very pleased to tell any nurse all I know about the Royal National Pension Fund for Nurses.

### A MATRON'S AUTHORITY.

"ANOTHER PROVINCIAL MATRON" writes: "In your issue of May 27th you say you do not think that there are many matrons who would resent the idea of a medical officer sending a nurse off duty for illness, without previously speaking to her, who is, you admit, the head of the nurses and of nursing. I venture to think that all who are not nonenties in their hospitals would resent such interference. Etiquette must be observed in institutions if friction is to be avoided, and the right thing for a medical officer to do in the event of his observing that a sister or nurse looked ill, would be to go to the matron's room and speak to her on the subject. For myself, I can say that I have never found my sisters and nurses backward in reporting their ailments to me, and I think that the matron who lives on the spot is quite as likely to notice any real illness as the medical officers. I am sure I should be exceedingly annoyed if my sisters came and told me that Dr. —— had sent Nurse A—— off duty; but I am thankful to say that the medical staff of this hospital are incapable of such discourtesy to me. It is I who report illness amongst the nurses to them and everything works smoothly. I am sure that you will of your courtesy publish this letter, though I differ from you.

### "THOMAS ATKINS" AS A HOSPITAL WORKER.

"A SISTER" of Queen Alexandra's Imperial Military Nursing Service of India, writes from Umballa: I feel I must acknowledge the courtesy of your manager in sending me a copy of THE NURSING MIRROR. I am not ordering it for the simple reason that, during all my Indian service (since 1891), I have taken in THE HOSPITAL, and shall hope to continue to do so always. We find the clinical articles most useful, so far away from lectures, etc., of any kind, and I often pass on my paper to a medical officer. Things are quite in a state of transition, and in many of our hospitals (including Umballa) tremendous advances have been recently made, bringing one's work more in line with one's home ideas. One surgical division is now quite distinct, and I find when training our men that they are quite keen on "sterilising," etc.—terms they had never even heard of in the old days. After nearly 12 years' experience, my opinion of "Thos. Atkins" as a hospital worker becomes higher and higher, and several of my old orderlies have taken up work when time-expired. One cavalry orderly corporal has obtained an excellent billet. My difficulties are:—1. A really suitable simple nursing book for the men. I often give them "Humphry Wards's," but, of course, that, and nearly all others, includes midwifery which I don't like them to have. 2. Names of institutions willing to enter on their books names of good orderlies wishing to take up the work when time-expired. I only recommend a man who has always been absolutely sober.

### AN OLD DODGE.

"R. D." writes: I was interested to read a nurse's account in your issue of May 20, having had a somewhat similar experience with a woman who answers to the description given in "Once bitten, twice shy." This person called upon me on a Monday morning, stating she had been recommended to come to me by a doctor in Sheffield whose name I knew, and engaged one of my best rooms for her sick sister-in-law. She gave me particulars of her sister-in-law's illness, who, she said, was now in Sheffield, where she herself lived, that she had spent the week end in Brighton, and wanted to make arrangements for her sister-in-law on her way through London. The sister-in-law was to be placed under the care of a well-known London specialist, who sends many patients to my house, and everything appeared to be perfectly genuine. Shortly before leaving my house, this person said that she regretted her purse and cards were in her dressing-bag at the station, as she would so much like to have bought one of the pretty blouses she had seen in the shop windows. Feeling assured that all was perfectly straightforward, I said, can I help you? She at first declined, but afterwards said she would be so glad, and accepted the loan of £2 10s., amply apologising for doing so, but promising to return it next day. She handed me a dirty card, which she said had her husband's name and address, Sheffield. She goes under the name of Mrs. Oxley; the address given was a fraud, and the supposed husband when written to replied that his wife never was in London, and neither of them knew the doctor mentioned in Sheffield, or the London doctor under whose care the supposed patient was to be placed.

### STATE REGISTRATION FEES.

"NURSE WITHOUT PRIVATE MEANS" writes: I can safely say that if I had to pay £5 5s. for registration (as Mrs. Bedford Fenwick suggests) I should be paying away more than half the money I own. I have already paid a 21 guinea premium and received a certificate for three years' nursing, having satisfied one set of examiners of a large general hospital. Therefore I should consider it hard lines to have to pay even three guineas for registration. As regards the co-operative system, as "A Sympathiser" suggests, if it were more general, there would be a chance of nurses saving. But thoroughly trained and competent nurses are bound to work for institutions at the salaries offered simply because there are so few co-operations, and those there are, if applied to, have "no vacancies at present" or "for months." I am not extravagant, and for long enough I have tried to save on a salary. But paying my way is about all I have succeeded in doing, and most nurses have calls upon their purse which cannot be overlooked. Another thing is that all co-operations are not like the Royal Chartered Nurses Association, the fees are not so good and the percentage is higher. All nurses cannot join that association if they would. I know of many fully trained and competent nurses who feel just the same.

MISS E. R. WORTABET writes: "As there seems some distress and indignation amongst some nurses, whilst others again have expressed a cheerfulness and willingness on the subject about my having suggested a fee of five guineas for State examinations and registration at the House of Commons, may I say that I think a profession is very cheaply bought at that price? But one of the members took me up for calling it a profession. Shall I call it a trade or a business? If so, what trade or what business or what means of livelihood can be bought at so small a price? Those of us who are fighting for State registration are not doing so for any selfish personal interest, but for the principle of the thing, for the good of the cause, and for the love of the profession for which we have given our minds, strength, money, and time. Each of us, perhaps, takes a different view of the subject, but we are all agreed on the lack of uniformity of training, and the fact of a three-years' certificate being no criterion that a nurse is "fully" trained—nurses so far being made a convenience of, and sisters of wards frequently being incapable of instructing or training probationers. State registration will rectify this wrong to nurses, as matrons will be compelled to divide the time for the different subjects and branches, so that a nurse on leaving a hospital will have gone through every department. I feel certain that when the public understand that nurses have to pass State examinations and pay a certain fee for registration the nursing profession will rise in dignity and value, and that a better class of women will be attracted. Lay nursing, as a profession for educated women, is one of the social evolutions and the most useful of this age. Having had experience in the training of English, French, and Syrian nurses, of nuns and deaconesses, I have sincerely come to the conclusion that educated women make the best nurses; piety, physical strength, and practical common sense are useful elements, but alone they are but broken reeds. I do not deny that half-educated women may make good, practical, useful nurses, but they always need the educated woman to think, to organise, to guide, and to command them. If the British nurse holds the position she does now in the professional world, if British hospitals are administered to the admiration of the world, if the British medical student acts and behaves as a gentleman in the hospital wards, it is thanks to the educated woman and to the position woman holds in Great Britain, and surely that woman can afford a fee of £5 5s. and can see for herself that State examinations and supervision will give weight and dignity, justice and equity to her profession.

NURSES AND MONEY-MAKING INSTITUTIONS.

"An Institution Matron" writes: Strongly-worded letters are perpetually being published regarding the iniquities of poor, unfortunate institutions which pay nurses a salary and percentage, and are sweepingly accused of "sweating" and other sorts of oppression by writers like "Sympathiser" in The Hospital. As one of these sinners I must say I object altogether to be classed among "sweaters," and I object also to the reflection such an assertion casts upon a body of sensible, skilled women like trained nurses, who are as free as air to do as they like, and cannot, and naturally would not, be "compelled" to submit to "sweating." That there are bad as well as good institutions everyone knows, but they are probably in the minority, and at any rate I only wish to speak for myself and from my own knowledge. Nurses have repeatedly told me that rather than incur the anxiety of working on their own account they much prefer the certainty of a settled salary with a comfortable home and no worrying about cases in slack times, which must come at intervals to everybody. Those whose circumstances or temperament cause them to think differently naturally work on their own account; but they each respectively do exactly as they like, and why shouldn't they? No one who has a living to make runs an institution or any other business on purely philanthropic lines, so "Sympathiser's" sneer about "money-making institutions" is out of place, and I would ask her to ponder for a few minutes on the liabilities, responsibilities, and anxieties involved, in carrying on an institution which may or may not "make money." Capital must be risked, and in its train are the rent, rates, taxes, salaries, percentage, uniforms, laundry, food, coals, gas, wages, telephone, repairs, renewals, bad debts, and all the hundred and one petty expenses which the nursing staff is spared. Surely after this the person upon whom this falls may be allowed to make a living out of her venture, or even "make money," albeit at the risk of "Sympathiser's" disapproval! Why should it be different from any other business in which women are engaged? And as to "sweating," one might as well call a doctor a "sweater" who has a salaried assistant who naturally does the coolie work and takes none of the fees. I have avoided mention of whether we make our nurses happy or not because, "blowing one's own trumpet" is objectionable; but I make the offer to "Sympathiser" that if she likes to communicate with me through the editor I will give her the opportunity of writing to any of our staff and asking them if they are "sweated" or otherwise oppressed. It is really time some reason was exercised in these uncalled-for attacks upon women as honourable and capable as—shall we say?—"Sympathiser" herself, but whose sin is that they try to earn an honest livelihood by supplying a public want and by giving employment to nurses in a way disapproved of by "Sympathiser." I was glad to see your note refuting that writer's argument that an agreement is not binding. The penalty clause is a common one in other businesses, but "Sympathiser" is perhaps ignorant of that, or only sees wickedness when it is applied to nursing institutions.

### Novelties for Nurses.

(By Our Shopping Correspondent.)

SOME EXCELLENT SOAPS.

Aberdeen has placed a hall-mark on many excellent commodities, and now I am able to speak in praise of soaps manufactured there, which are possibly little known to our readers. Most nurses like to keep by them a carbolic soap. The carbolic soap which has been sent from Aberdeen is a really reliable article, which is not always the case where an antiseptic is introduced. The coal tar is an equally good soap for the skin. Personally I have special words of praise for the delightful toilet soaps of Messrs. Ogston, which are superfatted and extra milled. They lather well and are most delicately perfumed. Amongst them is a nice transparent soap. Nurses will do well to secure specimens for themselves from the makers, Messrs. Ogston and Tennant, Soap Works, Aberdeen.

### Appointments.

[No charge is made for announcements under this head, and we are always glad to receive and publish appointments. The information, to insure accuracy, should be sent from the nurses themselves, and we cannot undertake to correct official announcements which may happen to be inaccurate. It is essential that in all cases the school of training should be given.]

Arbroath Burgh and District Joint Hospital.—Miss Isabella Keir has been appointed staff nurse. She was trained at the County Hospital, Motherwell, and has since been nurse at the Fever Hospital, Loanhead, Midlothian.

Colchester Hospital.—Miss Georgina Harvey has been appointed sister. She was trained at Crumpsall Infirmary, Manchester, and the Hospital for Women, Soho Square, London. She has since been charge nurse at the Cancer Hospital, Brompton, and sister at the Hospital for Diseases of the Chest, City Road, London.

City Fever Hospital, Little Bromwich, Birmingham.—Miss E. Moore has been appointed sister. She was trained at West Ham Infirmary, London, and Upney Fever Hospital, Barking, where she has since been charge nurse and deputy matron.

District Infirmary, Ashton-under-Lyne.—Miss Amy E. Thyra Scanlan has been appointed sister. She was trained at the Grimsby and District Hospital and the Hospital for Women, Soho, London. She has since been sister at West Ham Hospital, Stratford, and has also done district nursing in Sowerby Bridge, and Sedbergh, Yorkshire.

Dudley Workhouse Infirmary.—Miss Elizabeth Catherine Keefe has been appointed charge nurse. She was trained at Salford Union Infirmary, and has since been charge nurse at Holyhead Union Infirmary.

Francistown Hospital, Tati, Bechuanaland Protectorate.—Miss Elizabeth Fry has been appointed nurse-matron. She was trained at the Meath Hospital and County Infirmary, and afterwards did private and fever work in connection with the same institution. She has since been night-superintendent at Sheffield Royal Infirmary, has served as a member of the Army Nursing Service Reserve at Aldershot, and at Kimberley and Charlestown during the late war. She has for two years been nursing sister in the South African Constabulary.

Roxburgh District Asylum, Melrose. — Miss Annie McCloskey and Miss A. M. B. Keaghey have been appointed head nurse and night superintendent at the Roxburgh District Asylum, Melrose. Miss McCloskey was trained at the Royal Infirmary, Sheffield. She has since done private nursing, been in a surgical home at Glasgow, sister at Sheffield Royal Infirmary, and sister and night superintendent at the General Infirmary, Macclesfield. Miss Keaghey was trained at Meath Hospital, Dublin. She has since been Queen's nurse, and charge nurse at Craiglockhart Hospital, Edinburgh.

St. Mary (Islington) Infirmary, Highgate Hill, N.—Miss Annie F. Leroy has been appointed housekeeper. She was trained at University College Hospital and Queen Charlotte's Hospital, London. She has since been night superintendent and home sister at Bethnal Green Infirmary.

St. Pancras Infirmary, Hendon. — Miss Ethel Gladys Perrers has been appointed temporary sister for holiday-duty. She was trained at Southwark Infirmary, East Dulwich, and has since been ward sister at Bethnal Green Infirmary and charge nurse at the Eastern Hospital, Homerton.

Skipton Infectious Diseases Hospital.—Miss Pauline E. Allsop has been appointed sister. She was trained at St. Bartholomew's Hospital, London, and has since been temporarily out-patient sister at Nottingham Children's Hospital.

Wolverhampton Workhouse Infirmary.—Miss Annie Tyar has been appointed night sister in charge. She was trained at Southwark Infirmary, East Dulwich, and was afterwards charge nurse at the Eastern Fever Hospital, Homerton, and charge nurse and night superintendent at the Central London Sick Asylum.

### TRAVEL NOTES AND QUERIES.

By our Travel Correspondent.

Notice.—Those correspondents who are taking their holidays in August will not perhaps see answers to their questions directly in this column, because, owing to pressure on our space, we must attend to the wants of the early holiday makers first. But no one will be forgotten.

Model Cottages (F. B.).—Many thanks for information, will write later on.

Address of G.S.N.C. (Holiday in Belgium).—Address G.S.N.C., 33 Great Tower Street, E. Second-class return to Ostend 9s. Hôtel Grand Miroir, 56 Vieux Marché au Blé will suit you.

Brittany or Boulogne (A. M. W.).—See answer to "Normandie" in the issue of May 20th, and write me accordingly. The journey to St. Malo is dear, but to Belgium cheap. The answer to "A. M. D." will not suit you.

The English Lakes (Peggy).—See answer to "Daga" on May 20th. There are no cheap hotels. I should take a tourist ticket to the most central part of the district where you mean to take your holiday and leave yourself free and independent for small excursions.

A Week in a Convent (M. E.)—The place suggested would not suit you at all. If you would like a week in Belgium, *en route* for Germany, go to Knokke, reached by steam tram from Bruges. Terms extremely moderate at Hôtel de Bruges or Hôtel de la Marine. Or, if you prefer Bruges itself, go to Hôtel Panier d'Or in the Market Place. Terms from 6 francs.

Holiday at Ostend for Semi-invalid (H. H. H.).—Your question is such a difficult one that I must think it over and send an answer by post. You need not send 2s. 6d. I am sure the sufferer cannot afford it, and I shall be pleased to help her if I can. She must give up all idea of helping out her board by teaching; that is a plan very seldom adopted with success.

The Channel Islands (C. B.).—First-class return to Jersey £2 8s., second £1 17s. 6d. Second is quite comfortable. You need walk very little, a light railway and cheap coaches between them go everywhere all round the island. At Guernsey go to "The Crown Hotel." Terms from 5s. 6d. per day. You can also drive all round the island. Yes, it is cheaper to visit Sark in a day.

Thirty Shillings a Week Abroad (Kitty Grey).—Write to Mrs. Dyott, Villa Olga, St. Malo, France, and ask her if she will take you for that sum. Write on a prepaid return foreign letter-card. Your lack of French does not matter. St. Malo is very bracing, is a bright, gay little place, and surrounded by charming excursions. Journey, second-class return, from London, via Southampton, £2 1s. There is no railway at the other end.

Holiday in Scotland (F. N. B.).—You do not tell me what you can spend, so that I am in the dark. Such a tour as you propose would be very expensive. We do not undertake to give addresses of lodgings, and there are not many to be had where you wish to go. Moreover, lodgings are impossible when you change so often. A month spent seeing the places you speak of would cost, at the least, £14, exclusive of travelling. Let me hear further.

June in the Isle of Man (Cuckoo).—I am sorry to disappoint you, but I fear there is nothing to be had there at anything like your terms. The cheapest place I know of is 35s. per week. The only way would be for you to take a small room and cook and do for yourselves, but it would not be much holiday. The cheapest plan I can think of is to go to Belgium. Your return ticket would be 12s. and your board about £1. That is the best I can do for you. Let me know if you choose this at once.

A Week in Bruges for £5 (Old Maid).—Certainly you can do it for the money and you can stay longer. Go to Ostend by the General Steam Navigation Company's second class, return 9s., fare on to Bruges about 3s. return. I am sending you a private address by post where accommodation will suit you. Please to regard it as confidential. No, you must not think of Brussels, but you can visit Ghent, and Courtrai and Oudenarde from Bruges, also Ypres. Bruges itself is full of interest. Write to the manager of The Hospital for the issue of September 2nd, 1899. You will find an article of mine on Bruges which may save you the expense of a guide book.

Cornwall for Three Weeks (Decima).—Newquay is not a good centre; and it is not easy to get away from. If you are not tied to Cornwall I should suggest Lynton or Lynmouth in North Devon, from whence coaches seem to go everywhere. Lynton is on the top of the cliff, Lynmouth at the bottom. At Lynton write for terms to the "Queen's Hotel" and the "Crown." At Lynmouth to the "Rising Sun" and to Mr. Pedder, Postmaster, with whom you could board for 30s. a week. If you fix upon Cornwall I think Bude or Tintagel might do, but they have not the resources for excursions of the other places. At Tintagel there is a charming country inn, the "Wharncliffe Arms." At Bude try the "Globe." Another place you might like is Boscastle. Write there to Wivell's Private Hotel.

A Month's Holiday Abroad (Tired).—As you are determined to be by the sea, the convent is no good. Knokke is the place. Second-class return to Ostend by G.S.N.C., 9s. Fare on to Knokke by steam tram very trifling, perhaps 3 francs. Write to the Manager of The Hospital, enclosing three stamps, and ask him to send you The Hospital for September 1st, 1900. In an article of mine you will find all information. Hotel at Knokke, Grand Hôtel de Knokke, or Hôtel du Phare. These are close to the sea. Write and ask for terms; say you don't mind fourth floor, and wish to pay only 5 francs per day. This would come to 30s. a week. Still cheaper, but not quite so near the sea, Hôtel de Bruges. Write on a reply foreign letter-card. If this is still too much for you, I can tell you of something cheaper, but it would not be by the sea. The convent will not do, it is quite inland. So glad I have been able to help. Let me hear how you succeed. You can write to Knokke in English.

Zermatt for Four Ladies (Royal Oak).—I do not think you have sufficient money to do all you wish; it will not cover over three weeks' hotel expenses and so much travelling also. I think you are attempting too much. Second-class return to Zermatt for 45 days is £6 7s. 2d. via Dieppe. I do not think there is any cheaper independent arrangement. The cheapest place I know at Zermatt is Hot-Pension Bellevue, 6 to 7 francs. Accommodation is not cheap there, and all Pensions high up are more expensive. As to breaking your journey, it cannot, as you suppose, be done just anywhere. I think your wisest plan will be to write to Messrs. Cook, Ludgate Circus, telling them where you wish to go and asking the cheapest route. I am quite sure you cannot do all you propose; moreover, it will be very fatiguing. You propose to go nearly all round Switzerland. Do you know the Gemmi Pass is a very trying one to traverse? When you have heard from Cook I will try and help further.

Rules in Regard to Correspondence for this Section.—All questioners must use a pseudonym for publication, but the communication must also bear the writer's own name and address as well, which will be regarded as confidential. All such communications to be addressed "Travel Correspondent, 28 Southampton Street, Strand." No charge will be made for inserting and answering questions in the inquiry column, and all will be answered in rotation as space permits. If an answer by letter is required, a stamped and addressed envelope must be enclosed, together with 2s. 6d., which fee will be devoted to the objects of "The Hospital" Convalescent Fund. Ten days must be allowed before an answer can be published.

### Notes and Queries.

**REGULATIONS.**

**The Editor is always willing to answer in this column, without any fee, all reasonable questions, as soon as possible.**

**But the following rules must be carefully observed.**

1. **Every communication must be accompanied by the name and address of the writer.**
2. **The question must always bear upon nursing, directly or indirectly.**

**If an answer is required by letter a fee of half-a-crown must be enclosed with the note containing the inquiry.**

*Masseuse.*

(72) Will you kindly let me know through what institutions in London it is possible for a certified masseuse to get regular work? —*Nurse G.*

Write to the Secretary, the Incorporated Society of Trained Masseuses, 12 Buckingham Street, Strand, W.C.

*Nursing Sisterhoods.*

(73) Can you tell me where to obtain a list of the Church of England nursing sisterhoods, also any information concerning their work?—*Sister E.*

The Community of the Nursing Sisters of St. John the Divine is one. The address is 19 and 21 Drayton Gardens, South Kensington, London, S.W. Write to the Sister Superior for full particulars. The following institutions also belong to the Sisterhood:—St. John's Hospital, Morden Hill, Lewisham, S.E.; Convalescent Home, Seagray House, Littlehampton; and District Nursing Homes at Poplar and Deptford. A full list is given in "Burdett's Hospitals and Charities."

*Home.*

(74) Can you recommend any home for a lady who is suffering from rheumatism. Her general health is very good, but the rheumatism has settled in her legs and she is unable to walk. She cannot afford to pay a large sum, and if possible I should like to find a free home for her?—*F. S.*

See "Burdett's Hospitals and Charities" or "Homes and Hospitals for the Benefit of Gentlewomen," price 1s., from the Scientific Press.

*Children's Nurse.*

(75) I am anxious to go as children's nurse, and require training for same. I should be grateful if you could supply me with particulars where I could obtain the necessary training?—*A. M.*

If you mean that you wish to train as a nurse for sick children, see "The Nursing Profession: How and Where to Train." Otherwise write to the Secretary of the Norland Institute, Pembridge Square, London, W., or to the Honorary Secretary, "The Home," The Wrythe, Carshalton, Surrey.

*West Australia.*

(76) Can you tell me if there is a good chance of a nurse who has general training and C.M.B. certificate being successful in going out to West Australia to take up private nursing on her own account there.—*A. B.*

You would require capital to start on your own account and introductions.

*Nurses' Testimonials.*

(77) Can a lady superintendent of an institution or training school be compelled to give testimonials to her nurses?—*S. D.*

There is no compulsion, but it is usual for her to give either a testimonial or a reference.

*Sanatorium.*

(78) Will you kindly tell me where I could get three months' experience at a sanatorium for phthisis on the open-air system. I am a fully-trained nurse and have also experience in private and district work?—*Nurse H.*

You had better advertise or write to the matrons of the open-air sanatoria. For list apply, enclosing 7d., to the Scientific Press.

*Army Nursing.*

(79) Will you kindly tell me through your paper where I could get particulars regarding the Indian Military Nursing Service, and whether it is separate or in connection with the regular Army?—*C. W.*

Write for particulars to the Under-Secretary of State, India Office, St. James's Park, S.W.

**Handbooks for Nurses.**

| | Post Free. |
|---|---|
| "A Handbook for Nurses." (Dr. J. K. Watson.) ... | 5s. 4d. |
| "The Nurses' Dictionary of Medical Terms." ... ... | 2s. 0d. |
| "Massage" (Swedish.) ... ... ... ... | 2s. 6d. |
| "Surgical Ward Work and Nursing." ... ... ... | 3s. 10d. |
| "A Complete Handbook of Midwifery." (Watson.) ... | 6s. 4d. |

Of all booksellers or of The Scientific Press, Limited, 28 & 29 Southampton Street, Strand, London, W.C.

### For Reading to the Sick.

PRAYER.

"If we with earnest effort could succeed
To make our life one long connected prayer,
As lives of some perhaps have been and are,
If, never leaving Thee, we had no need
Our wandering spirits back again to lead
Into Thy Presence, but continued there,
Like angels standing on the highest stair
Of the sapphire throne, this were to pray indeed.
But if distractions manifold prevail,
And if in this we must confess we fail,
Grant us to keep at least a prompt desire,
Continual readiness for prayer and praise,
An altar heaped and waiting to take fire
With the least spark, and leap into a blaze."

*Trench.*

"I will lift up mine eyes unto the hills, from whence cometh my help. My help cometh from the Lord, which made heaven and earth."—*Psalm cxxi.*

No soul can preserve the bloom and delicacy of its existence without lonely musing and silent prayer: and the greatness of this necessity is in proportion to the greatness of the soul. There were many times during our Lord's ministry when, even from the loneliness of desert places, He dismissed His most faithful and most beloved, that He might be yet more alone.—*Archdeacon Farrar.*

It is a mistake to think that to serve God without feeling any pleasure in it is not pleasing Him; fresh roses are the most beautiful, but they have the most strength and fragrance when they are dry; so, though what we do for God is more agreeable to us when it is done with a lively tenderness of heart, because we judge of the pleasure that we feel, yet the fragrance of our actions is greater before God when they are done in a state of spiritual dryness. For then our will gives itself to the service of God in spite of all the repugnances which it has to overcome, and consequently it must have more strength and constancy than in a time of deeply felt devotion.—*S. Francis de Sales.*

Take care not to lay upon yourselves unnecessary burdens. Do not attempt more prayers than your time and strength allow. . . . Beware of a fidgetty, fussy kind of religion. Do not be over anxious. A great saint was once asked, "How can I live the higher life?" and he answered, "My child, go and live the lower life, and God will teach you the higher."—*Bishop Wilkinson.*

Yet he who at the sixth hour sought
The lone house top to pray,
There gained a sight beyond his thought—
The dawn of Gentile day.
Then reckon not, when perils lour,
The time of Prayer mis-spent;
Nor meanest chance, nor place, nor hour
Without its heavenward bent.

*Newman.*

